# State Plans for Containment of Pandemic Influenza

**DOI:** 10.3201/eid1209.060369

**Published:** 2006-09

**Authors:** Scott D. Holmberg, Christine M. Layton, George S. Ghneim, Diane K. Wagener

**Affiliations:** * Research Triangle Institute International, Atlanta, Georgia, USA;; † Research Triangle Institute International, Research Triangle Park, North Carolina, USA;; ‡ Research Triangle Institute International, Washington DC, USA

**Keywords:** Influenza, preventive measures, control, epidemiology, policy review

## Abstract

This review assesses differences and similarities of the states in planning for pandemic influenza. We reviewed the recently posted plans of 49 states for vaccination, early epidemic surveillance and detection, and intraepidemic plans for containment of pandemic influenza. All states generally follow vaccination priorities set by the Advisory Committee on Immunization Practices. They all also depend on National Sentinel Physician Surveillance and other passive surveillance systems to alert them to incipient epidemic influenza, but these systems may not detect local epidemics until they are well established. Because of a lack of epidemiologic data, few states explicitly discuss implementing nonpharmaceutical community interventions: voluntary self-isolation (17 states [35%]), school or other institutional closing (18 [37%]), institutional or household quarantine (15 [31%]), or contact vaccination or chemoprophylaxis (12 [25%]). This review indicates the need for central planning for pandemic influenza and for epidemiologic studies regarding containment strategies in the community.

## Abstract 2

Current plans for control of pandemic influenza vary, and many do not include nonpharmaceutical interventions.

Much recent attention, public, governmental, and academic, has been focused on the possibility of an influenza pandemic, possibly arising from a mutated or genetically reassorted strain of the currently circulating avian influenza virus (H5N1). In the United States, state and local health departments are primarily responsible for detecting an outbreak and implementing the public health response. Accordingly, individual states and the US Department of Health and Human Services (HHS) have each recently released pandemic influenza plans and guidelines. These plans are now available (Table A1). Because state health departments are autonomous of federal control, their approaches to surveillance and containment are likely to vary. Thus, this review was undertaken to determine the extent of differences between states in their approaches to detecting and controlling pandemic influenza.

## State Procedures and Plans

Forty-nine states have Internet websites that include statewide pandemic influenza procedures and plans or, in a few instances, have simply addressed broad questions about state-based responses to pandemic influenza (Table A1). Often these plans, which are funded by a Centers for Disease Control and Prevention (CDC) preparedness cooperative agreement, were posted in the last half of 2005 or early 2006, and these documents are still largely in transition. US HHS has recently issued guidelines (*1*); however, specific planning, problem solving, and funding are still left to the individual states.

Almost all of the states' plans address a wide range of issues regarding command and control, surveillance, vaccination, antiviral drugs, communication, and emergency management and containment measures. The purpose of this review was to focus on community public health strategies, especially vaccination, surveillance and detection, and containment, which the various states develop as they refine their plans.

## General Processes

### Vaccination

In general, all 49 states with posted plans are in accordance with one another on vaccination priority strategies (Table A2). Many state plans indicated that they were constrained by the uncertainties of future vaccine and antiviral drug supply and effectiveness and the properties of a future influenza epidemic.

Nonetheless, general agreement exists, explicit or implicit, to provide vaccination during a pandemic that is prioritized by those most likely to acquire, become ill, or die from pandemic influenza (Table A2). Generally, these guidelines were based on Advisory Committee on Immunization Practices (ACIP) recommendations (*2*) and HHS guidelines (*1*) if the latter were available at the time the state plan was drafted. The clear priorities involved the following groups: 1) healthcare workers (some states also include emergency responders such as emergency medical technicians, fire fighters, and police officers; political leaders; utility workers; transporters of vaccine; and vaccine manufacturers); 2) persons with respiratory, immunodeficiency, cardiovascular or other high-risk conditions; 3) persons >65 years of age (17 [35%] plans also consider vaccinating children 6–23 months of age); 4) household members, caregivers, and other close contacts of those ill or likely to be ill; and 5) all others.

The first 3 of the these groups were variously prioritized by the states as high-priority vaccine recipients, but all states consider these 3 groups as the highest priority. A few states, such as Maine, have estimated that 15%–20% of their populations would be in such high-priority groups to be vaccinated.

### Surveillance and Detection

Of the 49 states that have posted their plans, all rely on the National Sentinel Physician Surveillance (NSPS), the nationwide 122 Cities Mortality Reporting System of pneumonia- and influenza-related deaths (*3**,**4*), or both. Forty-seven (96%) states also explicitly indicated they would evaluate reports of clusters or unusual influenzalike cases by local physicians, clinics, and institutions in their state, and 40 states (82%) indicated they would evaluate reports of laboratory-confirmed influenza from state health department and other laboratories (Table A2).

Although many states describe plans for enhanced surveillance during pandemic influenza, relatively few (12 [25%]) currently have or envision real-time syndromic surveillance of influenzalike illness (ILI) in persons seeking care at clinics or hospital emergency departments to detect the onset of pandemic influenza (Table A2).  To our knowledge, the cities currently using syndromic surveillance are New York City, Washington, DC, Pittsburgh, and some cities in North Carolina and Virginia. In addition, the CDC BioSense program (*5*) plans to expand syndromic surveillance to 300 clinical sites by the end of 2006.

Eight states (16%), including California (especially in Los Angeles), New York and Hawaii, are developing ways of screening incoming international travelers. However, current plans for other international entry points, such as Seattle, Portland, Chicago, and Atlanta, do not indicate any similar (foreign) international traveler quarantine and testing activities.

### Containment Measures

The various state plans are markedly heterogeneous in their personal contact-avoidance measures and prophylaxis (Table A2). Most states outline pandemic influenza responses that do not include general and early encouragement of many specific personal avoidance steps, such as staying home from work and keeping sick children at home.

Seventeen (35%) states explicitly plan or are considering recommending (voluntary) personal social isolation on the community level, such as staying at home or keeping children at home if they feel sick. Eighteen (37%) other states cite federal or state regulations that indicate that health authorities may close schools, businesses, and other institutions during a severe outbreak, although they are not required to resort to such closures. However, 15 (31%) other states also indicate legal ability to quarantine persons, households, or institutions. (However, in some states such as Michigan, the efficacy of quarantine is directly questioned in their current document.)

Given the high cost and limited supply of neuraminidase inhibitor antiviral drugs and an uncertain supply and effectiveness of future vaccines, only 12 (25%) states plan or consider using either chemoprophylaxis (such as oseltamivir) or vaccination of household and other close nonhospital contacts in their plans to retard epidemic influenza.

## Implications

The control of future pandemic or interpandemic influenza will necessarily rely on each individual state's plan to vaccinate persons and detect and contain this disease. Still, the current national (HHS) pandemic influenza plan presents only a categorization and listing of steps, rather than explicit direction for the states. This lack of central coordination can result in a patchwork of plans that will not adequately detect and control this or other respiratory disease pandemics.

Given the lack of clear guidance, coupled with the fact that no one knows when an influenza pandemic may strike, what its characteristics will be, and the effectiveness and quantity of strain-specific vaccine, the evolving state plans are nonetheless in agreement in adhering generally to ACIP and HHS guidelines for prioritizing vaccination (*1**,**2*). In general, the elderly, those with chronic diseases, and healthcare and infrastructure personnel will be prioritized to receive vaccination, and in approximately one third of states, young children will be prioritized to be vaccinated (*6*). We believe the estimate that such persons make up ≈15%–20% of the population in any state is reasonable (*1*). However, this vaccination strategy is predicated on preventing deaths from influenza, not stopping or retarding an epidemic or pandemic (*7*). Given that vaccine adequate to cover the entire US population will not be available for several more years, the goal of reducing transmission would require much more vaccination than is available.

Regarding surveillance and detection, state plans are even more variable than they are about strategizing vaccinations. All states indicate that they plan to use the NSPS network and the nationwide 122 Cities Mortality Reporting System (*3**,**4*). In NSPS, each year ≈1,100 (55%) of ≈2,000 healthcare providers nationwide voluntarily report the number of weekly outpatient visits for ILI and submit specimens from a subset of patients to state public health laboratories for influenza virus testing. The 122 city-specific mortality reporting is unavoidably even more delayed. Neither system would likely detect a local outbreak of influenza <2 weeks into its establishment. Thus, some state and federal authorities are assessing the utility of syndromic surveillance of ILI (e.g., cough, headache, and fever) in emergency departments and clinics in test cities. Several such programs exist, and CDC's BioSense (*5*) includes plans to expand such early surveillance to 300 clinical sites. However, to our knowledge, no health authority feels confident that earlier detection of influenza by 1 to 3 weeks would necessarily lead to better control or substantial retardation of an outbreak.

Finally, confusion and lack of specificity exist in these posted state plans in proposing practical containment measures in the community. The national HHS Pandemic Influenza Plan (*1*) has several recommendations for infection control in the hospital setting but is weaker and nonspecific in other areas such as control of influenza in the community. For example, there is no agreed-upon definition of geographic clustering of cases or number of persons infected that will trigger the declaration of a pandemic. Much of this national plan suggests social distancing and respiratory (cough) etiquette and devotes much of its discussion to mask use. Accordingly, states are comparably nonspecific about community control plans. Vaccination or chemoprophylaxis of contacts is infrequently addressed, mainly because the vaccine supply is limited, as are the most effective antiviral drugs, the neuraminidase inhibitors oseltamivir and zanamivir, for the next several years. Even when these vaccines and drugs become available, considerable obstacles remain to detecting influenza promptly and getting vaccination or drug therapy to the contacts of influenza patients.

Several practical nonpharmaceutical containment steps need to be considered. For example, only approximately one third of the state plans are explicitly considering recommending self-isolation of adults with influenzalike symptoms and keeping children with such symptoms home from school and daycare. Even in this increasingly computer-based economy, in which a considerable percentage of persons can work from home most of the time, this simple stratagem is not addressed in most state plans. Other simple recommendations for use in the community, such as avoiding mass gatherings; shopping on off hours; and household and workplace strategies such as frequent hand washing, avoiding handshaking, and keeping towels separate, are often neglected in state plans.

Why are there these state plans so disparate? We believe some of the problem results from weak central (federal) direction, as has been a criticism of national bioterrorism preparedness (*8*). Fortunately, state and federal plans are still in flux, many are still in draft form (Table A1), and getting a clearer delineation of a basic plan that all states can follow is still possible. The US Secretary of HHS has been meeting with states (http://www.pandemicflu.gov/plan/convening.html) to review pandemic preparedness issues.

However, we also believe that answers are lacking to several key epidemiologic questions necessary for rational planning. What is the typical intrahousehold or institutional attack rate, and would vaccination or chemoprophylaxis of contacts retard or stem outbreaks? How well do masks work for pandemic influenza in the community, and when and for how long should they be used? Does closing a school or other institution actually reduce community-level illness and death? Does earlier detection of influenza in a community lead to behavior changes that could stem an outbreak? We know of no studies designed to address these and several other issues; e.g., the Models of Infectious Disease Agents Study (MIDAS) (*9*) has been forced to rely on estimates of household attack rates estimated >30 years ago (*10**,**11*) for a nonpandemic (H3N2) strain of influenza virus. Several state plans, such as California's, expressed frustration about this lack of information.

Thus, we believe that a revision of the national pandemic influenza plan, which despite unavoidable gaps in our knowledge, relies on professional and public health opinion to provide more uniform, specific, and practical influenza protection, avoidance and containment practices for pandemic and interpandemic influenza in the community would be helpful. We also believe it would be prudent to begin studies and, in the interim, create expert panels to determine if masks, school closings, social isolation, and several other nonpharmaceutical strategies would be useful in reducing the illness and death caused by pandemic influenza and its spread in the community.

## Appendix

**Table A1:** Source documents of state pandemic influenza plans

State	Website	Date
US overview	http://www.hhs.gov/pandemicflu/plan/	4 Nov 2005
	http://www.pandemicflu.gov/plan/stateplans.html	
Alabama	http://www.adph.org/IMMUNIZATION/AL_PI_Plan_100305.pdf	4 Oct 2005
Alaska	http://www.epi.hss.state.ak.us/id/influenza/fluinfo/pandemicfluplan.pdf	Mar 2005 (draft)
Arizona	http://www.azdhs.gov/pandemicflu/index.htm	Aug 2005
Arkansas	http://www.healthyarkansas.com/pandemic_influenza/pandemic_influenza_plan.pdf	2 Aug 2005 (revised)
California	http://www.dhs.ca.gov/ps/dcdc/izgroup/pdf/pandemic.pdf	18 Jan 2006 (draft)
Colorado	http://www.cdphe.state.co.us/bt/HealthProviders/PandemicPlanDraft.pdf	30 Sep 2005 (draft no. 2)
Connecticut	http://www.dph.state.ct.us/BCH/infectiousdise/pdf/vol25no4_fnlclr.pdf	Nov 2005 (state epidemiology letter)
Delaware	http://www.dhss.delaware.gov/dhss/dph/files/depanfluplan.pdf	12 Sep 2005 (final)
Florida	http://www.doh.state.fl.us/disease_ctrl/epi/htopics/flu/Pandemicdraft8.pdf	Mar 2004 (draft)
Georgia	http://health.state.ga.us/pdfs/epi/GafluPandemicPrepPlan.pdf	Oct 2005 (revised)
Hawaii	http://www.hawaii.gov/health/about/reports/pandemicflu.pdf	26 Oct 2005 (pending plan) (fact sheet)
Idaho	http://www.cste.org/specialprojects/Influenzaplans/IDHWPandemicInfluenzaPreparednessandResponsePlan1102.pdf	22 Oct 2004 (year 1 draft)
Illinois	http://www.idph.state.il.us/flu/Pandemic_Flu_Plan_3_17_06.pdf	17 Mar 2006 (revised)
Indiana	http://www.in.gov/isdh/pdfs/PandemicInfluenzaPlan.pdf	23 Aug 2005
Iowa	http://www.protectiowahealth.org/documents/Exec_Summary_Pandemic_Annex.pdf	4 Nov 2005
Kansas	http://www.kdheks.gov/flu/download/KS_Pan_flu_10_05.pdf	Oct 2005
Kentucky	http://chfs.ky.gov/dph/epi/preparedness/pandemicinfluenza.htm	Mar 2006
Louisiana	Not available	
Maine	http://www.maine.gov/dhhs/boh/DRAFT%20Pan%20Flu%20Plan%20071205_revised_rb.doc	22 Aug 2005 (draft)
Maryland	http://edcp.org/pdf/THEPLAN3READ.pdf	Apr 2002 (version 5)
Massachusetts	http://www.mass.gov/dph/cdc/epii/flu/statepln.pdf	5 Oct 2005 (revised)
Michigan	http://www.michigan.gov/documents/Pandemic_v2_142241_7.00.12.pdf	15 Nov 2005 (version 2.1)
Minnesota	http://www.hsem.state.mn.us/readyminnesota/New_Ready_Web/PanFluSup.pdf	8 May 2006
Missouri	http://www.dhss.mo.gov/PandemicInfluenza/PandemicPlan.pdf	9 Feb 2006
Mississippi	http://www.msdh.state.ms.us/msdhsite/index.cfm/44,1136,122,154,pdf/SNSPlan.pdf	Mar 2005
Montana	http://www.dphhs.mt.gov/PHSD/Communicable-disease/pdf/Annex4-Pandemic-Flu-Plan-7.14.05.pdf	6 Jul 2005
Nebraska	http://www.hhs.state.ne.us/pandemic_guidelines.pdf	4 Nov 2005 (draft)
Nevada	http://health2k.state.nv.us/administration/Influenza/Notices/PandemicFluPlan.pdf	11 Nov 2005 (fact sheet)
New Hampshire	http://www.dhhs.nh.gov/NR/rdonlyres/eug6g37griemk6m3fmu3cuwrzgm6qlquo5ovi4jglw4bni3yehfojgrrpseggwf7d4wzhrh2z7eibymnpaahwkwdoib/FLU_pandemic+plan+version+3-2-06.pdf	2 Mar 2006 (interim plan)
New Jersey	http://www.state.nj.us/health/flu/documents/draft.pdf	1 Jul 2005
New Mexico	http://www.cste.org/specialprojects/Influenzaplans/NMPandemicfluplan10-5-2004.pdf	Aug 2005 (draft)
New York	http://www.health.state.ny.us/diseases/communicable/influenza/pandemic/docs/pandemic_influenza_plan.pdf	7 Feb 2006
North Carolina	http://www.epi.state.nc.us/epi/gcdc/pandemic.html	Jan 2006
North Dakota	http://www.health.state.nd.us/EPR/resources/PandemicInfluenzaPlanSummary.pdf	9 Mar 2006
Ohio	http://www.odh.state.oh.us/ASSETS/399540667CF446508D5E663610B7396A/ODHFluPlan.pdf	Sep 2005 (version 2.0)
Oklahoma	http://www.health.ok.gov/program/cdd/flu/Oklahoma%20PIM%20Plan%20Final%20WEB%20DRAFT.pdf	1 Jul 2005
Oregon	http://www.cste.org/specialprojects/Influenzaplans/OregonPandemicFlu2005.pdf	Jan 2005
Pennsylvania	http://www.dsf.health.state.pa.us/health/lib/health/flu/PandemicFluInfo2005.pdf	Jul 2005 (revised)
Rhode Island	http://www.health.ri.gov/flu/index.php	Nov 2005 (fact sheet)
South Carolina	http://www.scdhec.net/administration/ophp/pandemic_preparedness.htm	Mar 2006
South Dakota	http://www.state.sd.us/doh/Flu/SD%20Flu%20Pandemic%20Draft%20Plan%20Oct-05.pdf	20 Oct 2005 (draft)
Tennessee	http://www2.state.tn.us/health/Ceds/PDFs/2006_PanFlu_Plan.pdf	Jul 2006
Texas	http://www.cste.org/specialprojects/Influenza%20Pandemic%20State%20Plans/TxPanFluPlan1.15.2004.pdf	15 Jan 2004
Utah	http://health.utah.gov/epi/diseases/flu/ClinicianPublicHealth/pandemic/pandemic_influenza_plan.pdf	2 Nov 2005
Vermont	http://healthvermont.gov/panflu/docs/draft-Pan-Flu-Plan3.3.pdf	23 Jan 2006
Virginia	http://www.vdh.state.va.us/PandemicFlu/pdf/DRAFT_Virginia_Pandemic_Influenza_Plan.pdf	Mar 2006
Washington	http://www.doh.wa.gov/panflu/plansummary.htm	Nov 2005 (webpage: general information)
West Virginia	http://www.wvflu.org/PDFs/wv_strategy_overview_for_summit.pdf	Dec 2005
Wisconsin	http://dhfs.wisconsin.gov/preparedness/pdf_files/WIPandemicInfluenzaPlan.pdf	Apr 2004
Wyoming	http://wdh.state.wy.us/epiid/wpirp.pdf	Aug 2005

**Table A2:** Overview of 50 state plans for vaccination, surveillance, and containment of pandemic influenza*. PDF Available at www.cdc.gov/eid/pdfs/06-0369-TA2.pdf (131 KB, 2 pages)

State	Vaccination		Surveillance and detection				Community containment activities considered			
	ACIP priority groups	Other groups considered†	Clinics/hospitals/clinicians	Laboratories	Syndromic surveillance‡	International travelers	Voluntary self-isolation	School/institution closings	Institution/household quarantine	Contact vaccination/chemoprophylaxis
Alabama	Yes		Yes	Yes						Possible
Alaska	Yes		Yes	Yes						
Arizona	Yes		Yes	Yes			Yes			
Arkansas	Yes	Children 2–18 y of age	Yes		Nonprescription medicine use		Yes	Yes	Yes	
California	(Yes)	(Yes)	Yes	Yes	Yes	Yes	Yes	Yes	Yes	Yes
Colorado	Yes		Yes	Yes						
Connecticut	Yes		Hospitals only	Yes						
Delaware	Yes	Yes	Yes	Yes						Yes
Florida	Yes	Community service perssonnel† and preschoolers	Yes	Yes				Possible		Yes
Georgia	Yes		Yes	Yes					Possible	
Hawaii	Yes		Yes	Yes		Yes	Yes	Possible	Possible	
Idaho	Yes		Possible	Yes	Possible		Possible	Possible		
Illinois	(Yes)		Yes		Possible					
Indiana	Yes	Yes	Yes	Yes			Yes	Possible	Yes	Yes
Iowa	(Yes)		Yes	(Yes)		(Yes)				
Kansas			Yes	Yes		(Yes)	"Will follow generic…response plans.."			
Kentucky	Yes	Yes	Yes	Yes						(Yes)
Louisiana	No pandemic influenza plan posted before Hurricane Katrina									
Maine	Yes;		Yes	Yes		(Yes)	Yes	Possible	Yes	
Maryland	Yes		Yes	Yes		(Yes)				
Massachusetts	Yes	Yes	Yes	Yes			Yes	Yes		Yes
Michigan	Yes	(Yes)	Yes	Yes		Possible	Yes	Yes		
Minnesota	Yes		Yes	Yes	Yes, in development					
Mississippi	(Yes)		Yes	(Yes)						
Missouri	Yes	Yes	Yes	Yes						
Montana	Yes		Yes	Yes						
Nebraska	(Yes)		Yes	Yes	Yes, in development				Possible	
Nevada	(Yes)		Yes	Yes			Yes			
New Hampshire	Yes		Yes	Yes				Yes		
New Jersey	Yes		Yes	Yes			Yes	Yes	Yes	(Yes)
New Mexico	Yes		Yes	Yes			(Yes)	(Yes)		
New York	(Yes)	(Yes)	Yes	Yes	Yes	Yes	Yes	Yes	Yes	(Yes)
North Carolina	Yes	(Yes)	Yes	Yes	Yes		(Yes)	(Yes)	(Yes)	Possible
North Dakota	Yes	(Yes)	Yes	Yes						
Ohio	Yes		Yes	Yes				Possible	Possible	
Oklahoma	Yes		Yes	Yes				Possible	Possible	
Oregon	Yes		Yes	Yes						
Pennsylvania	(Yes)		Yes		Pittsburgh		Yes		Possible	
Rhode Island	Yes									
South Carolina	Yes	(Yes)	Yes				(Yes)			
South Dakota	Yes	(Yes)	Yes	(Yes)			(Yes)			(Yes)
Tennessee	(Yes)		Yes							
Texas	(Yes)	(Yes)	Yes	Yes	Yes, in development				Yes	
Utah	Yes		(Yes)					Possible		
Vermont	Yes	(Yes)	Yes	Yes						(Yes)
Virginia	(Yes)		Yes	Yes	Yes					
Washington	(Yes)		(Yes)							
West Virginia	Yes	(Yes)								
Wisconsin	Yes		Yes	Yes	Possible					
Wyoming	Yes		Yes	Yes				(Yes)	(Yes)	

*ACIP, Advisory Committee on Immunization Practices; (Yes), a program was explicitly considered but not finalized in the state plan; Possible, a program was alluded to but not extensively considered. †Other groups include nuclear power plant workers, telecommunications and public utility workers, and key government personnel. ‡Syndromic surveillance refers to the immediate electronic collection and reporting of patients in clinics, emergency departments, and other medical venues who have cough, fever, and other symptoms suggestive of influenza.
